# Modified citrus pectin ameliorates methotrexate-induced hepatic and pulmonary toxicity: role of Nrf2, galectin-3/TLR-4/NF-κB/TNF-α and TGF-β signaling pathways

**DOI:** 10.3389/fphar.2025.1528978

**Published:** 2025-01-23

**Authors:** Randa Ismail, Heba A. Habib, Aliaa F. Anter, Amr Amin, Gehan H. Heeba

**Affiliations:** ^1^ Department of Pharmacology and Toxicology, Faculty of Pharmacy, Minia University, El-Minia, Egypt; ^2^ College of Medicine, University of Sharjah, Sharjah, United Arab Emirates

**Keywords:** methotrexate, modified citrus pectin, galectin-3, hepatotoxicity, lung toxicity

## Abstract

**Introduction:**

Methotrexate (MTX) is a frequently utilized anti-inflammatory and anticancer agent. Its potential liver and lung toxicity often limits its clinical effectiveness. We conducted this study to demonstrate the possible protective impacts of a natural galectin-3 (Gal-3) inhibitor, modified citrus pectin (MCP), against MTX-induced liver and lung toxicity and verify the potential signaling pathways of these suggested effects. *In vitro*, the cytotoxicity of MCP and its modulatory effect on MTX cytotoxic efficacy were assessed.

**Methods:**

Four groups of rats were used: control, MTX (40 mg/kg, single intraperitoneal injection on day 9), MTX + MCP (200 mg/kg/day, orally, for 2 weeks), and MCP alone. MCF7, Nalm6, and JEG3 cell lines were used for the *in vitro* cytotoxicity assay.

**Results:**

MCP counteracted liver and lung toxicity evidenced by ameliorating the markers of liver and lung functions. Moreover, MCP minimized oxidative stress elicited by MTX in lung and liver tissues, as indicated by reduced malondialdehyde levels, elevated levels of reduced glutathione, increased superoxide dismutase activity, and upregulated Nrf2 protein expression. In hepatic and pulmonary tissues, MCP downregulated the inflammatory signaling pathway, Gal-3/TLR-4/NF-κB/TNF-α. MCP pretreatment decreased TGF-β, collagen content, and cleaved caspase-3 levels. MCP enhanced the cytotoxicity of MTX in Nalm6 and JEG3 and did not interfere with its cytotoxicity in the MCF7 cell lines.

**Discussion:**

MCP attenuated MTX-induced liver and lung toxicity through antioxidant, anti-fibrotic, anti-inflammatory, and anti-apoptotic influences, as demonstrated by the improved histopathological changes induced by MTX in pulmonary and hepatic tissues. Moreover, it increased MTX cytotoxicity in different human cell lines.

## 1 Introduction

Methotrexate (MTX), a folate antagonist, is a competitive inhibitor of dihydrofolate reductase which converts dihydrofolate into tetrahydrofolate required for nucleic acid synthesis (Kremer, 2004; Koźmiński et al., 2020). However, it is used in high doses for managing various cancers such as acute lymphoblastic leukemia, osteosarcoma, and breast cancer (Ezhilarasan, 2021). Furthermore, it is used for gestational choriocarcinoma (Chin, 2023). Low-dose MTX is used as an anti-inflammatory and immunomodulatory drug as the first line and centerpiece therapy for rheumatoid arthritis and as maintenance therapy for Crohn’s disease and psoriasis (Pivovarov and Zipursky, 2019; Hsieh and Tsai, 2023). Unfortunately, however, adverse effects associated with MTX use such as gastrointestinal toxicity, hepatotoxicity, nephrotoxicity, pulmonary toxicity, and neurotoxicity limit its clinical application (Triantafyllou et al., 2010; Mahmoud et al., 2017b). Steatohepatitis, fibrosis, as well as cirrhosis, are forms of liver toxicity triggered by MTX (Romão et al., 2014). Among MTX-treated patients, the prevalence of hepatic fibrosis and cirrhosis was up to fifty percent and twenty-six percent, respectively (Roghani et al., 2020). Meanwhile, MTX’s acute hypersensitivity pneumonitis and interstitial lung inflammation which usually appear within the first year of therapy occur in approximately 8% of patients with an estimated mortality of 13%–17% (Romão et al., 2014). Moreover, lung fibrosis can also occur with MTX (Juge et al., 2021).

The pathogenesis of MTX-induced hepatotoxicity and lung damage is not well clarified. However, it was documented that oxidative stress is the critical factor, as it can initiate an inflammatory response and oxidative DNA damage, inducing apoptotic cell death (Mahmoud et al., 2017b; Chauhan et al., 2020; Ozmen et al., 2024). Furthermore, the profibrotic effects of MTX were identified in both liver and lung tissues (Mohamed et al., 2019). MTX activates fibroblasts, which then induce extracellular matrix synthesis, leading to tissue fibrosis and organ dysfunction (Fayez et al., 2018). That is why finding therapeutic agents that can be used with MTX to reduce the incidence and severity of its associated adverse effects is sought after.

Galectin-3 (Gal-3) is a multifunctional mammalian β-galactoside-binding lectin that is expressed on the cell surface, nucleus, cytoplasm, and extracellularly. It is mainly secreted by macrophages and participates in numerous biological events, for example, cell adhesion, migration, angiogenesis, and apoptosis (Nangia-Makker et al., 2000; Henderson et al., 2008; Li et al., 2014). Its key role in tissue inflammation and fibrosis was documented. Gal-3 activation in different fibrotic models in addition to abnormally elevated levels in patients who have liver, lung, or heart fibrosis have been reported (Nishi et al., 2007; Bayes-Genis et al., 2014; Sciacchitano et al., 2018).

Modified citrus pectin (MCP) is obtained from citrus fruit as a water-soluble dietary fiber. It is a natural inhibitor of Gal-3 by binding directly to its carbohydrate recognition domain (Glinsky and Raz, 2009; Gunning et al., 2009). Recently, MCP gained popularity due to its anti-cancer (Glinsky and Raz, 2009; Leclere et al., 2013; Garrido et al., 2024), anti-inflammatory, and antifibrotic effects (Kolatsi-Joannou et al., 2011; Abu-Elsaad and Elkashef, 2016; Li et al., 2018; Marín-Royo et al., 2018; Xu et al., 2020; Cui et al., 2022; Bouffette et al., 2023) in several diseases. It showed hepatoprotective effects against carbon tetrachloride (CCl4)-caused liver fibrosis in rats through antioxidant and Gal-3 blockade-mediated antifibrotic and antiapoptotic effects (Abu-Elsaad and Elkashef, 2016). Nevertheless, the involvement of Gal-3 inhibition in MTX-elicited liver and lung illnesses is still unclear.

For the first time, our goal was to discover the role of Gal-3 in the pathogenesis of MTX-induced hepatotoxicity and lung toxicity and the possible protective effects of its natural inhibitor, MCP. We also explored the potential signaling pathways that could explain these suggested preservative effects. Finally, different human cancer cell lines were used here to analyze the impact of MCP on the cytotoxic effect of MTX as an *in vitro* part of the study.

## 2 Materials and methods

### 2.1 Drugs

Methotrexate was procured from the Austrian pharmaceutical company EBEWE Pharma, Ges.m.b.H. Nfg. KG. MCP (Pectasol) was obtained from EcoNugenics, Santa Rosa, CA, U.S.A.

### 2.2 Animals

2 Here, we procured adult male albino Wistar rats (180–220 g) from Helwan Farm in Cairo, Egypt, owned by Vacsera Company. Animals had free access to water and food throughout the 2 weeks of the acclimatization period and the experiment period. They were subjected to a 25°C ± 2 temperature and 12:12 h of dark/light cycles. With the approval number: MPEC-230506, the present study protocol complied with the regulations set out by the Research Ethics Committee at the Faculty of Pharmacy at Minia University in Egypt.

### 2.3 Experimental design

After randomly dividing the rats into four groups of eight, they were administered the following dosage schedule:

1. Control group: Rats were given distilled water (MCP vehicle) orally for 14 days and a single intraperitoneal saline (MTX Diluent) injection on day 9.
2. MCP group: Rats were administered MCP (200 mg/kg/day, orally for 14 days) and a single intraperitoneal saline injection on day 9.
3. MTX group: Rats received distilled water orally for 14 days and a single intraperitoneal MTX injection (40 mg/kg) on day 9.
4. MTX + MCP group: Rats were given MCP (200 mg/kg/day, orally for 14 days) and a single intraperitoneal MTX injection (40 mg/kg) on day 9.

The dosage and timing for MCP were determined based on our preliminary studies and prior pharmacological investigation (Li et al., 2021) which showing its protective impact against organ damage. Meanwhile, the MTX dose was chosen to be sufficient to elicit hepatic and pulmonary injury in male rats (Letertre et al., 2020; Dogra et al., 2021; Matouk et al., 2022; Matouk et al., 2023). MTX injection on day 9 was done based on prior research (Matouk et al., 2022). Furthermore, our preliminary study results indicated that MCP showed hepatic and pulmonary protective effects when given 200 mg/kg/day for 8 days before giving MTX on day 9.

### 2.4 Blood and tissue sampling

Rats were put under anesthesia 24 h following the final dose. A cardiac puncture was made to take samples of blood in clean centrifuge tubes and then centrifuged at 3,500 rpm for 10 min to get sera which were used freshly for liver function assessment. The liver and lungs were rapidly isolated and weighed after drying on filter paper. Relative liver and lung weights were calculated (Relative organ weight = ((organ weight/body weight) × 100) (Jakkula et al., 2000; Wan et al., 2021). Immunohistochemical and histopathological examinations were conducted on parts of the lower lobe of the right lung and the medial lobe of the liver after fixing them in 10% formalin. Before the biochemical analysis, the residual liver parts and left lung were kept at −80°C after being quickly frozen in liquid nitrogen.

### 2.5 Evaluation of microvascular permeability and lung edema

Rapidly after blood collection by cardiac puncture, we obtained the bronchoalveolar lavage fluid (BALF) through tracheal intubation and lavage of both lungs with 2 mL saline. The BALF samples underwent centrifugation at 1,000 rpm at 4°C for 10 min (Rajizadeh et al., 2024). The supernatant was used to measure the BALF total protein spectrophotometrically using a commercially available kit (BioMed, Cairo, Egypt) according to Kingsley (1939). Meanwhile, total leukocyte count was detected by Mindray Bc-20 s Auto Hematology analyzer after resuspending the cell pellet in 0.5 mL phosphate buffer saline (PBS) (Zhao et al., 2021). To assess the wet/dry (W/D) weight ratio as an indicator of lung edema, the right upper lobe weight was determined alone after lung separation, wet weight. Then 24 h of drying was done in an oven at 80°C, dry weight (Zhang et al., 2021).

### 2.6 Determination of liver function markers

The serum alanine aminotransferase (ALT) and aspartate aminotransferase (AST) levels were assessed using commercial kits purchased from Biodiagnostic, Cairo, Egypt as described by Reitman and Frankel (1957).

### 2.7 Liver and lung histopathology

After fixation of the liver and lung sections in 10% formalin, they were embedded in paraffin blocks after dehydration with graded ethanol series, cleared with xylene, and then sectioned into 4–6 µm thickness slices. For histopathological examination, deparaffinization and hematoxylin and eosin (H&E) staining of the produced sections were performed following Bancroft and Gamble (2008). The damage was evaluated according to the scoring system by Plaa et al. (1994) in the liver and by Eldh et al. (2012) in the lung. Moreover, Masson’s trichrome staining was done to evaluate organ fibrosis. Fibrosis was assessed semi-quantitatively in ×200 magnification. The area percentage (%) of tissue with fibrotic changes on Masson’s trichrome-stained sections was evaluated using ImageJ software (Van De Vlekkert et al., 2020).

### 2.8 Assessment of oxidative stress parameters in liver and lung homogenates

Lipid peroxidation in hepatic and lung homogenates was analyzed as thiobarbituric acid reactive species (TBARS) named malondialdehyde (MDA) employing the methodology established by Buege and Aust (1978). Reduced glutathione (GSH) content and superoxide dismutase (SOD) activity have been investigated as markers of endogenous antioxidant defense. Ellman (1959) methodology was used to measure GSH content. Whereas, SOD activity was assessed following Marklund (1985).

### 2.9 Assessment of Nrf2, TLR-4, NF-κB, and c-caspase-3 using western blot analysis

Hepatic and pulmonary tissues were homogenized using a protease inhibitor cocktail (Biospes, China) and tris lysis buffer for 30 min at 4°C following the method described by Ali et al. (2018). After that, centrifugation at 10,000 rpm for 10 min at 4°C was done to remove residual tissue. Total protein concentrations were assessed using the Biuret method Wang et al. (1996). Utilizing 10% sodium dodecyl sulfate-polyacrylamide gel electrophoresis, equivalent quantities of protein, 30 μg of total protein for each lane, underwent electrophoresis and then transferred to a polyvinyl difluoride membrane (Millipore, USA) applying semi-dry transfer methods (Towbin et al., 1979). To block the membranes, they were incubated for 60 min at room temperature with 5% non-fat milk in tris-buffered saline Tween 20.

Next, the membranes were incubated with the primary antibodies for the target proteins overnight at 4°C; toll-like receptor-4 (TLR4) (dilution 1:1,000) (Santa Cruz Biotechnology, Inc., sc-293072), nuclear factor erythroid 2-related factor 2 (Nrf2) (dilution 1:500), nuclear factor-kappa B (NF-κB p65) (dilution 1:500), cleaved caspase-3 (c-caspase-3) (dilution 1:500) (Chongqing Biospes Co., Ltd., China, YPA1865, BBP1066, and YPA2210, respectively) and β-actin (dilution 1:3,000) (Elabscience Biotechnology, Inc., E-AB-20031). The membranes were mixed with an alkaline phosphatase-conjugated secondary antibody (dilution 1:5000) for 1 hour obtained from Biospes, China. Band visualization was achieved by BCIP/NBT substrate detection Kit obtained from Genemed Biotechnologies, United States of America. Analysis of the produced bands compared to the internal control β-actin bands was conducted utilizing ImageJ^®^ software (National Institutes of Health, Bethesda, Maryland, United States of America).

### 2.10 Assessment of TNF-α using enzyme-linked immunoassay (ELISA) technique

A rat TNF-α ELISA kit was used to assess tumor necrosis factor-alpha (TNF-α). It employs the sandwich ELISA principle, wherein samples were added to precoated microwells with TNF-α specific antibody. The microplate was then incubated with avidin-horseradish peroxidase (HRP) conjugate and biotinylated antibody. Substrate solution was added, causing a blue color. A stop solution was used to end the reaction, turning blue to yellow. At 450 nm, the optical density was measured which is directly proportional to the TNF-α concentration.

### 2.11 Assessment of Gal-3 and TGF-β using immunohistochemistry

After deparaffinizing and rehydrating the liver and lung tissue sections, they were soaked in hydrogen peroxide and washed in buffer to inhibit peroxidase activity. Non-specific background staining was blocked using Ultra V block. Antibodies targeting Gal-3 (Novocastra laboratories, UK, clone 9c4) and transforming growth factor-beta (TGF-β1) (Chongqing Biospes Co., Ltd., China, YPA1196) were incubated with liver and lung tissue sections exactly as directed by the manufacturer. Afterward, they were incubated at room temperature for 10 min with a primary antibody enhancer and then for 15 min with HRP polymer. Hematoxylin was used as a counterstain to contrast the chromogen color (Shan et al., 1999).

Immunoreactivity was assessed semi-quantitatively in high microscopic power fields (X400). The area percentage (%) of positively stained cells was evaluated by using Fiji ImageJ software (Schindelin et al., 2012).

### 2.12 *In vitro* analysis

Cell culture was done at Vacsera-cell culture laboratory, Cairo, Egypt. The cytotoxicity assay was evaluated on the tested drugs using breast cancer (MCF7), acute lymphoblastic leukemia (Nalm6), and choriocarcinoma (JEG3) cell lines. The American Type Culture Collection in Manassas, Virginia, United States of America was the source of all the cell lines. These cells were grown in RPMI 1640 media provided with 10% fetal bovine serum, 1% penicillin, and 1% streptomycin, and then incubated with 5% CO_2_ at 37°C.

### 2.13 Assessment of the cytotoxic effect of MCP and MTX using MTT assay

An *in vitro* toxicological assay kit (Sigma Aldrich, Inc., M-5655) which is MTT-based was utilized to assess MTX, MCP, and their combination cytotoxicity on MCF7, Nalm6, and JEG3 cell lines. The cancer cells were seeded into a 96-well plate containing 100 μL of the culture media at 1 × 10^4^ cells/mL density and incubated for 1 day. Afterwards, the culture media was exchanged with 100 μL of a new media with different concentrations of the tested drugs; MTX and MCP (0.4, 1.6, 6.3, 25, 100 μg/mL) and vehicle control, 0.01% dimethyl sulfoxide (DMSO), for 48 h. Each well of the plates was incubated for another 4 hours with 20 μL of MTT in 200 μL of media. Eliza microplate reader (BDR206, Bioline Technology, India) was used for optical density determination, with a 570 nm absorbance. Each concentration of each drug was done in triplicate wells for assessment of IC50 (the concentration required to inhibit 50% of cell growth) of MTX and MCP. After the assessment of the IC50 of each drug for each cell line, another experiment was carried out. The IC50 of MTX was added in combination with that of MCP to the three types of cancer cells for measuring the percentage of cell viability of each cell line to study the effect of MCP on MTX cytotoxicity.

### 2.14 Statistical analysis

Results representation was done as mean ± standard error of the mean (SEM). One-way analysis of variance (ANOVA) was performed to figure out the statistically significant differences. Thereafter, the comparison between the means of all groups was done using the Tukey-Kramer post-analysis test. A *P*-value below 0.05 was deemed significant by using Version 9.00 for Windows of GraphPad Prism^®^ (GraphPad Software, United States of America, https://www.graphpad.com/).

## 3 Results

### 3.1 Effect of MTX and MCP on relative liver weight and liver function biomarkers

Following the MTX intoxication, the relative liver weight was significantly (*P* < 0.05) elevated compared to the control group while the MTX-induced hepatomegaly was significantly (*P* < 0.05) alleviated by coadministration of 200 mg/kg of MCP (Table 1). Moreover, MTX led to a significant (*P* < 0.05) elevation in serum levels of ALT and AST in comparison to the control group. In contrast to the MTX group, MCP treatment significantly (*P* < 0.05) mitigated the increased serum AST and ALT levels (Table 1).

**TABLE 1:** Effect of MTX and MCP on relative liver weight and liver function biomarkers.

Groups	Relative liver weight	Serum ALT (U/mL)	Serum AST (U/mL)
Control	2.69 ± 0.06	79.51 ± 1.33	73.25 ± 4.07
MCP	2.82 ± 0.01	80.44 ± 1.04	87.69 ± 4.17
MTX	3.20 ± 0.02*	106.70 ± 2.69*	188.50 ± 3.73*
MTX + MCP	2.79 ± 0.14^#^	67.70 ± 4.23^#^	107.20 ± 5.52^#^

Data are expressed as mean ± SEM., Significant differences compared to the control and MTX, groups were denoted by * and #, respectively, at P < 0.05. MTX: methotrexate; MCP: modified citrus pectin; AST: aspartate transaminase; ALT: alanine transaminase.

### 3.2 Effect of MTX and MCP on relative lung weight, lung W/D weight ratio, BALF total protein, and leukocyte count

In contrast to the control group, the relative lung weight and lung W/D weight ratio, indicators of pulmonary edema, were significantly (*P*< 0.05) increased in the MTX group. Of interest, both relative lung weight and lung W/D weight ratio were attenuated significantly (*P*< 0.05) with MCP treatment compared to the MTX group (Table 2).

**TABLE 2:** Effect of MTX and MCP on relative lung weight, lung W/D weight ratio, BALF total protein, and leukocyte count.

Groups	Relative lung weight (mg/g)	Lung W/D weight ratio	Total protein (g/dL) in BALF	Total leukocytes in BALF (*10^3^)
Control	6.21 ± 0.16	6.92 ± 0.18	1.43 ± 0.08	2.76 ± 0.11
MCP	5.93 ± 0.35	6.85 ± 0.19	1.53 ± 0.09	3.02 ± 0.06
MTX	8.56 ± 0.40*	8.97 ± 0.31*	3.28 ± 0.04*	4.57 ± 0.14*
MTX + MCP	6.14 ± 0.19^#^	7.11 ± 0.28^#^	1.50 ± 0.08^#^	2.74 ± 0.12^#^

Data are expressed as mean ± SEM., Significant differences compared to the control and MTX, groups were denoted by * and #, respectively, at P< 0.05. MTX: methotrexate; MCP: modified citrus pectin; BALF: bronchoalveolar lavage fluid; W/D weight ratio: Wet/Dry weight ratio.

Table 2 illustrates that the MTX intoxication produced a significant (*P*< 0.05) rise in total leukocyte count and total protein content in BALF, markers of inflammation, compared to the control group. Co-treatment with MCP significantly (*P*< 0.05) decreased both total leukocyte counts and total protein content in BALF compared to the MTX alone.

### 3.3 Effect of MCP on histopathological changes in hepatic and pulmonary tissues induced by MTX

**FIGURE 1:**
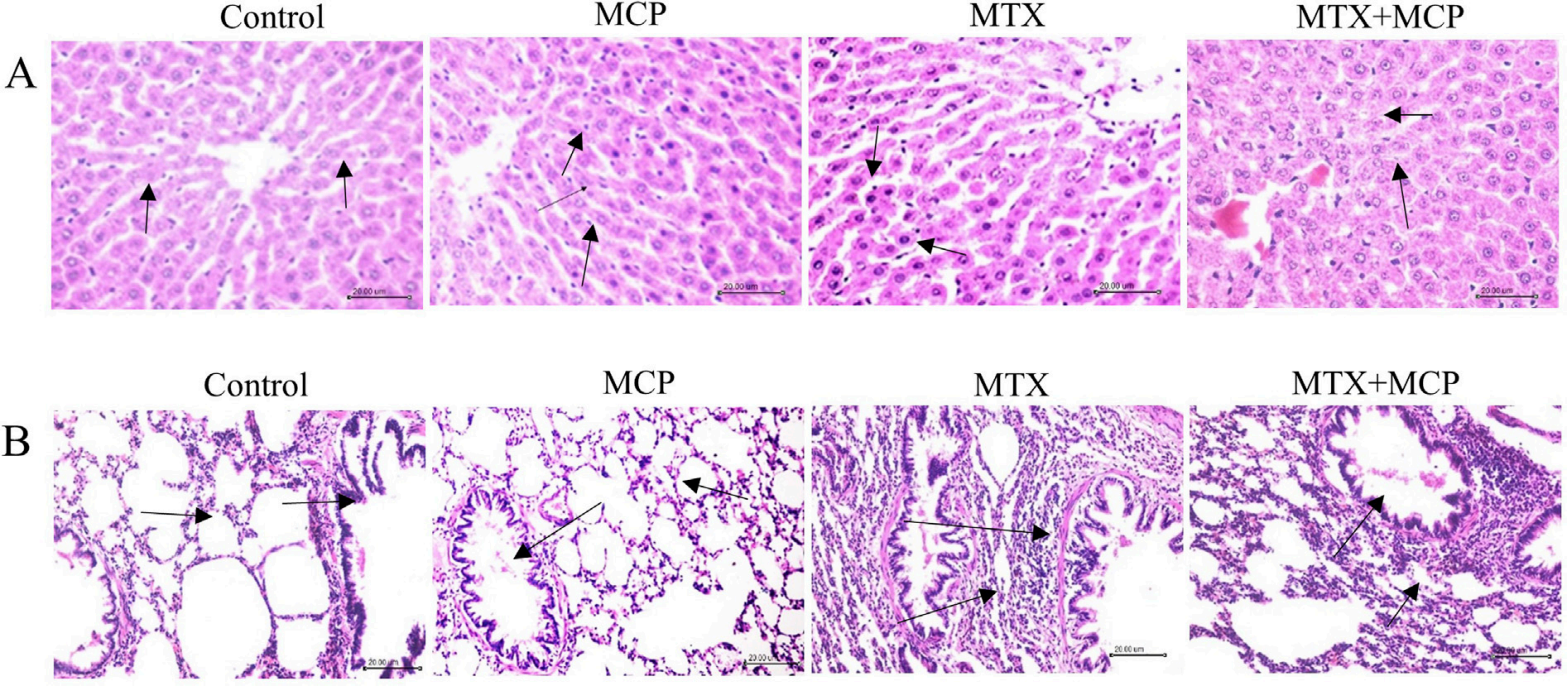
Hematoxylin and eosin staining of liver and lung tissue sections. **(A)** Representative photomicrographs of hepatic tissue sections (X200) (scale bar = 20 µm). Liver sections of the control and MCP groups show an obvious central hepatic vein and well-organized hepatic cords (arrow). The liver section from the MTX group shows hepatocyte apoptosis and nuclear pyknosis (arrow), and the liver section from the MTX + MCP group exhibits narrowed liver sinusoids and hepatocyte ballooning degeneration (arrow). **(B)** Representative photomicrographs of lung tissue sections (X200) (scale bar = 20 µm). Pulmonary sections of the control and MCP groups show the normal structure of the alveoli (arrow). The lung tissue section of the MTX group displays thickened alveolar walls and focal collapsed areas with inflammatory cell infiltration of the interstitium (arrow). The lung tissue section of the MTX + MCP group shows mild thickened inter-alveolar septa and focal emphysematous regions (arrow). MTX: Methotrexate; MCP: Modified citrus pectin.

Regarding H&E staining, Figure 1A demonstrates hepatic tissue sections of the control and MCP groups at which hepatic lobules exhibit normal histological architecture. Well-organized hepatic cords with polygonal hepatocytes interconnected in anastomosing plates, with borders facing either the neighboring hepatocytes or the sinusoids were also observed (grade 0).

Conversely, the hepatic tissue sections of the MTX group displayed disorganized hepatic cords. Kupffer cells hyperplasia and narrowed hepatic sinusoids were observed. Moreover, hepatocyte ballooning degeneration, accompanied by nuclear pyknosis, and apoptosis, manifested as intense scattered eosinophilic bodies throughout the hepatic lobules, were also indicated (grade IV). The hepatic tissue sections of MTX-intoxicated animals treated with MCP showed mild hepatocyte swelling having granular cytoplasm and central vesiculated nuclei with peripheral chromatin condensation. Kupffer cells hyperplasia and narrowed hepatic sinusoids were also observed (grade I).

Regarding lung sections, the lung tissues obtained from the control and MCP groups revealed lung lobules with normal histological architecture. The alveoli were delineated with inter-alveolar septa and blood capillaries with tiny connective tissue surrounding these blood vessels (score 0). In the MTX group, the lung tissue exhibited widespread inflammatory cellular infiltration, primarily macrophages and lymphocytes, congested blood capillaries, and markedly thickened inter-alveolar septa. Moreover, bronchial goblet cells were absent. Numerous areas of focal collapse accompanied by the formation of giant alveoli were also noticed (score 4) as shown in Figure 1B.

Contrarily, the pulmonary tissue sections of the MTX + MCP group demonstrated moderate inflammatory cell infiltration. Mild thickened inter-alveolar septa with numerous focal emphysematous regions (score 2) were also observed (Figure 1B).

**FIGURE 2:**
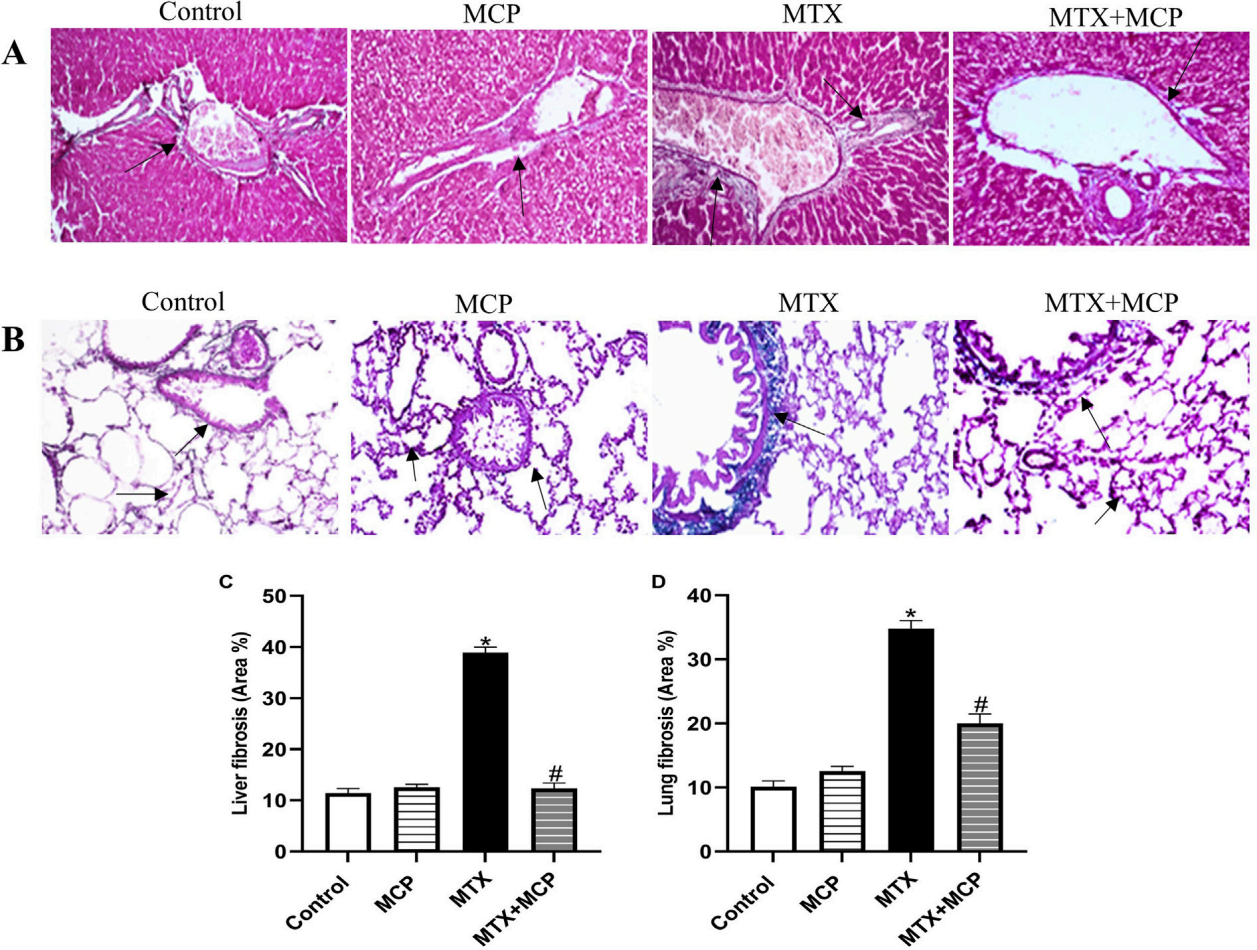
Masson’s trichrome staining of liver and lung tissue sections. **(A)** Representative photomicrographs of hepatic tissue sections (X200). Liver sections of the control and MCP groups show normal portal triad morphology with delicate fibrous tissue (arrow). The liver section from the MTX group shows fibrous tissue proliferation with hyperplasia of the bile duct (arrow), and the liver section from the MTX + MCP group exhibits the normal histological structure of portal triad (arrow). **(B)** Representative photomicrographs of lung tissue sections (X200). Pulmonary sections of the control and MCP groups show delicate fibers in some alveolar and bronchial walls (arrow). The lung tissue section of the MTX group shows a peribronchial fibrotic thickening arrow (arrow). The lung tissue section of the MTX + MCP group shows gentle fibrotic changes of the alveolar and bronchial wall (arrow). **(C)**: The bar chart represents the semi-quantitative analysis of the area percentage of liver tissue with fibrotic changes on Masson’s trichrome-stained liver sections of control, MCP, MTX, and MTX + MCP groups. **(D)**: The bar chart represents the semi-quantitative analysis of the area percentage of lung tissue with fibrotic changes on Masson’s trichrome-stained lung sections of control, MCP, MTX, and MTX + MCP groups. Data are represented as mean ± SEM. *, # refer to significant differences compared to the control and MTX groups, respectively*, at* P < 0.05. MTX: Methotrexate; MCP: Modified citrus pectin.

Concerning Masson’s trichrome staining, the liver tissue sections of the control and MCP groups exhibited normal morphology of the portal triad consisting of a branch of the portal vein, hepatic artery, and bile duct supported by delicate fibrous tissue. Conversely, the liver tissue section of the MTX group showed fibrous tissue proliferation with bile duct hyperplasia. On the other hand, the liver tissue section of the MTX + MCP group revealed a normal distribution of the fibrous tissue with a dilated portal vein (Figure 2A). Semi-quantitative analysis of liver fibrosis exhibited a significant (*P* < 0.05) elevation in fibrosis in the MTX group compared to the control group. On the other hand, the MTX + MCP group had a significant (*P* < 0.05) decline in liver fibrosis compared to the MTX group (Figure 2C).

Masson’s trichrome-stained lung tissue sections of the control and MCP groups revealed delicate fibers in some alveolar and bronchial walls. In contrast, the MTX group showed fibrotic thickening of alveolar septa, peribronchial, and periarteriolar regions. Contrarily, the lung tissue section of MTX + MCP displayed gentle fibrotic changes in the alveolar and bronchial walls (Figure 2B). Semi-quantitative analysis of lung fibrosis revealed a significant (*P* < 0.05) increase in lung fibrosis in the MTX group compared to the control group. On the other hand, the MTX + MCP group had a significant (*P* < 0.05) decrease in lung fibrosis compared to the MTX group (Figure 2D).

### 3.4 Effect of MCP on MTX-caused oxidative stress in hepatic and pulmonary tissues

**FIGURE 3:**
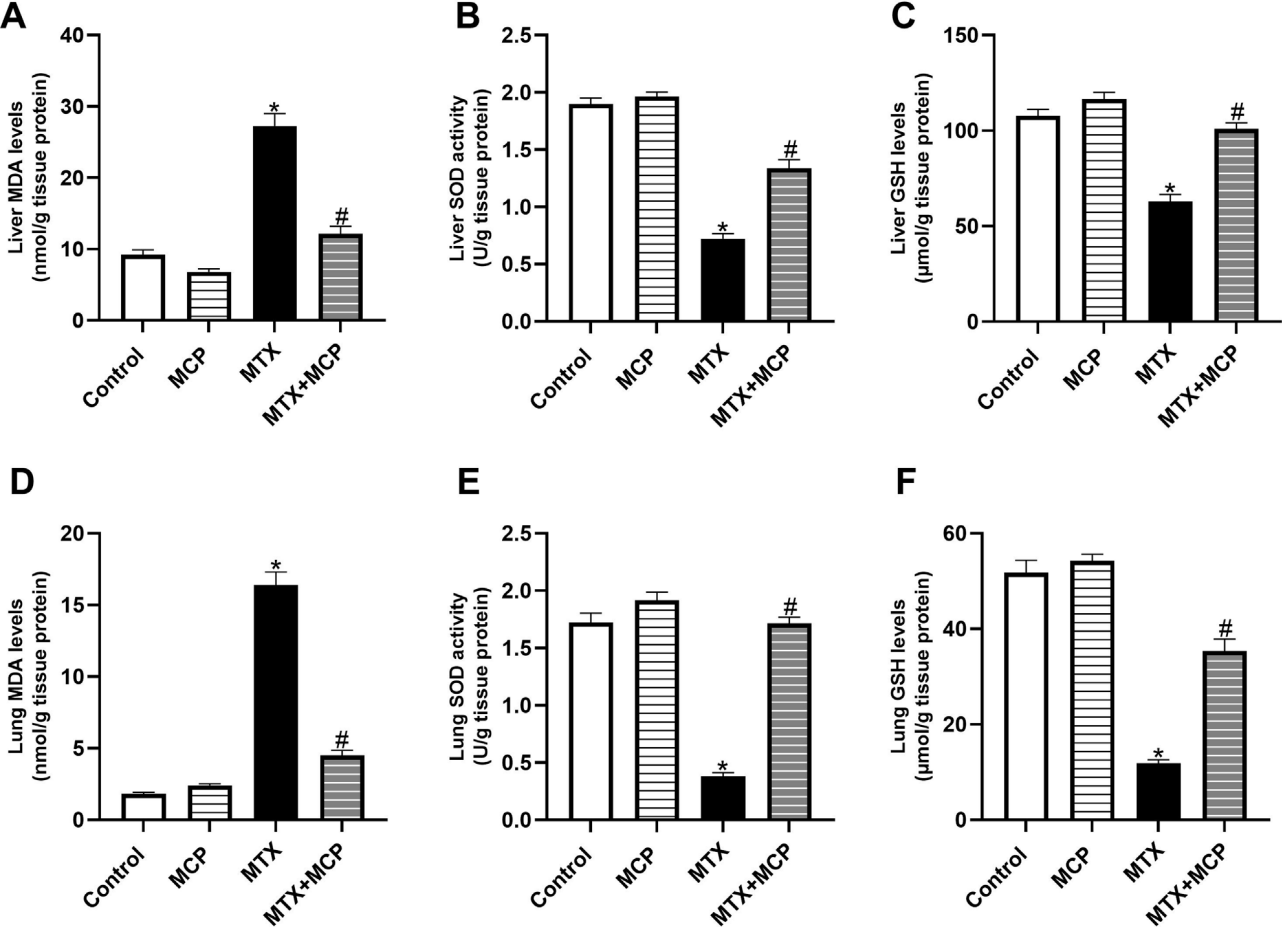
Effect of MCP on MTX-caused oxidative stress in liver and lung tissues. **(A)** Liver MDA level, **(B)** Liver SOD activity, **(C)** Liver GSH level, **(D)** Lung MDA level, **(E)** Lung SOD activity, and **(F)** Lung GSH levels. Data are expressed as mean ± SEM. Significant differences compared to the control and MTX groups were denoted by * and #, respectively, at P<0.05. MTX: Methotrexate; MCP: Modified citrus pectin; MDA: malondialdehyde; GSH: Reduced glutathione; SOD: Superoxide dismutase.

Methotrexate administration resulted in a significant (*P*< 0.05) elevation in MDA levels in hepatic and pulmonary tissues compared to the control group (Figures 3A, D). On the contrary, co-administration with MCP significantly (*P* < 0.05) reduced the MDA levels.

Consistent with MDA findings, a significant (*P* < 0.05) reduction in SOD activity and GSH content in both liver (Figures 3B, C) and lung tissues (Figures 3E, F) was detected with MTX administration compared to the control group. The impairment in the endogenous antioxidant capacity was significantly (*P* < 0.05) hindered by pre-conditioning with MCP.

### 3.5 Effect of MTX and MCP on Nrf2 expression in hepatic and pulmonary tissues

**FIGURE 4:**
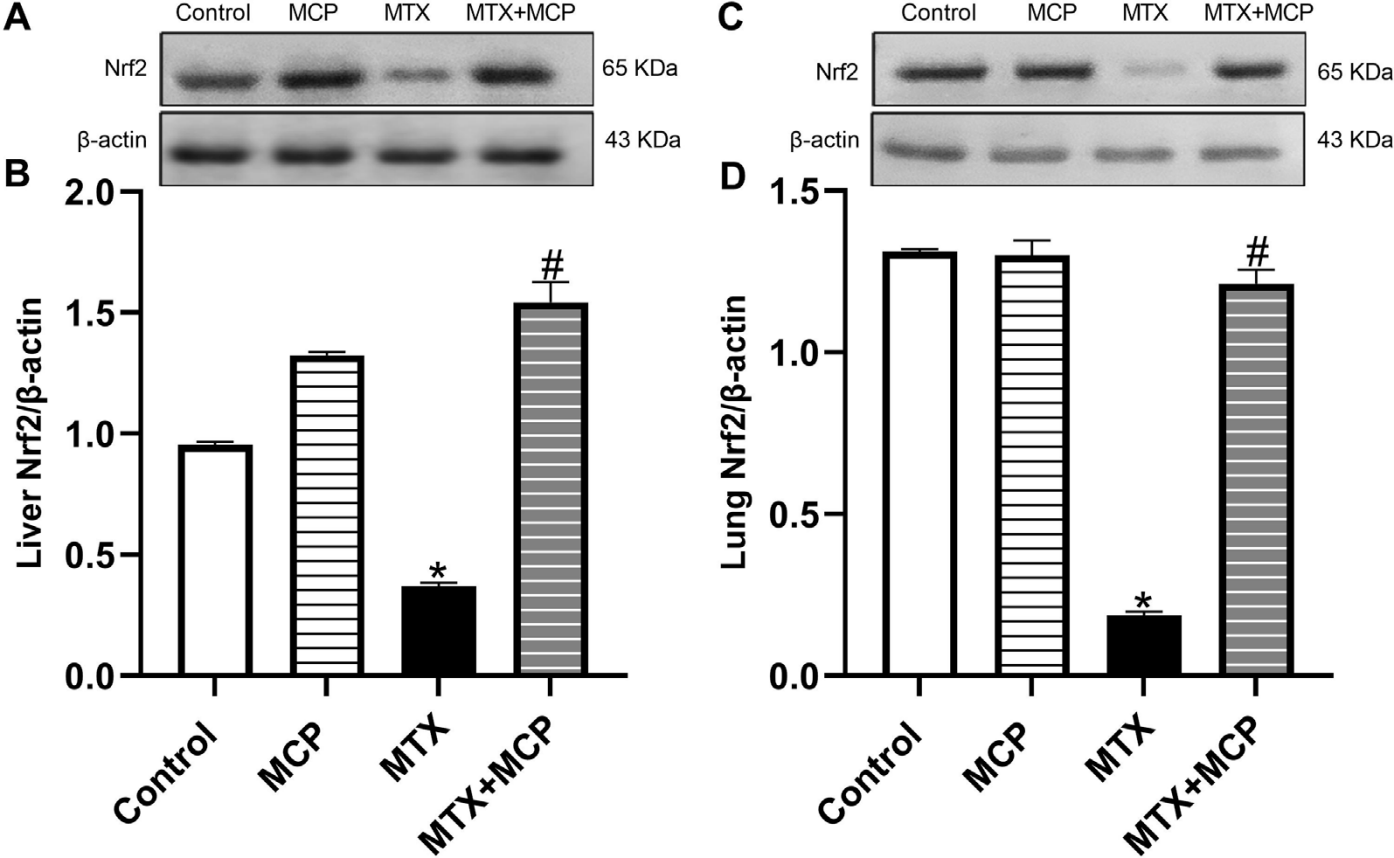
Western blot analysis of the effect of MTX and MCP on Nrf2 expression in hepatic and pulmonary tissues. **(A, C)** represent the Nrf2 bands of control, MCP, MTX, and MTX + MCP groups of liver and lung tissues, respectively. **(B, D)** represent the bar charts of semi-quantitative densitometric analysis of Nrf2 bands in both liver and lung tissues, respectively. Data are expressed as mean ± SEM. Significant differences compared to the control and MTX groups were denoted by * and #, respectively, at P < 0.05. MTX: Methotrexate; MCP: Modified citrus pectin; Nrf2: Nuclear factor erythroid 2-related factor 2.

Compared to the control group, MTX significantly (*P* < 0.05) diminished Nrf2 protein expression in liver and lung tissues, while co-treatment with MCP significantly (*P* < 0.05) prevented this decrease induced by MTX, as shown in Figure 4.

### 3.6 Effect of MTX and MCP on Gal-3 immunoreactivity in liver and lung tissues

**FIGURE 5:**
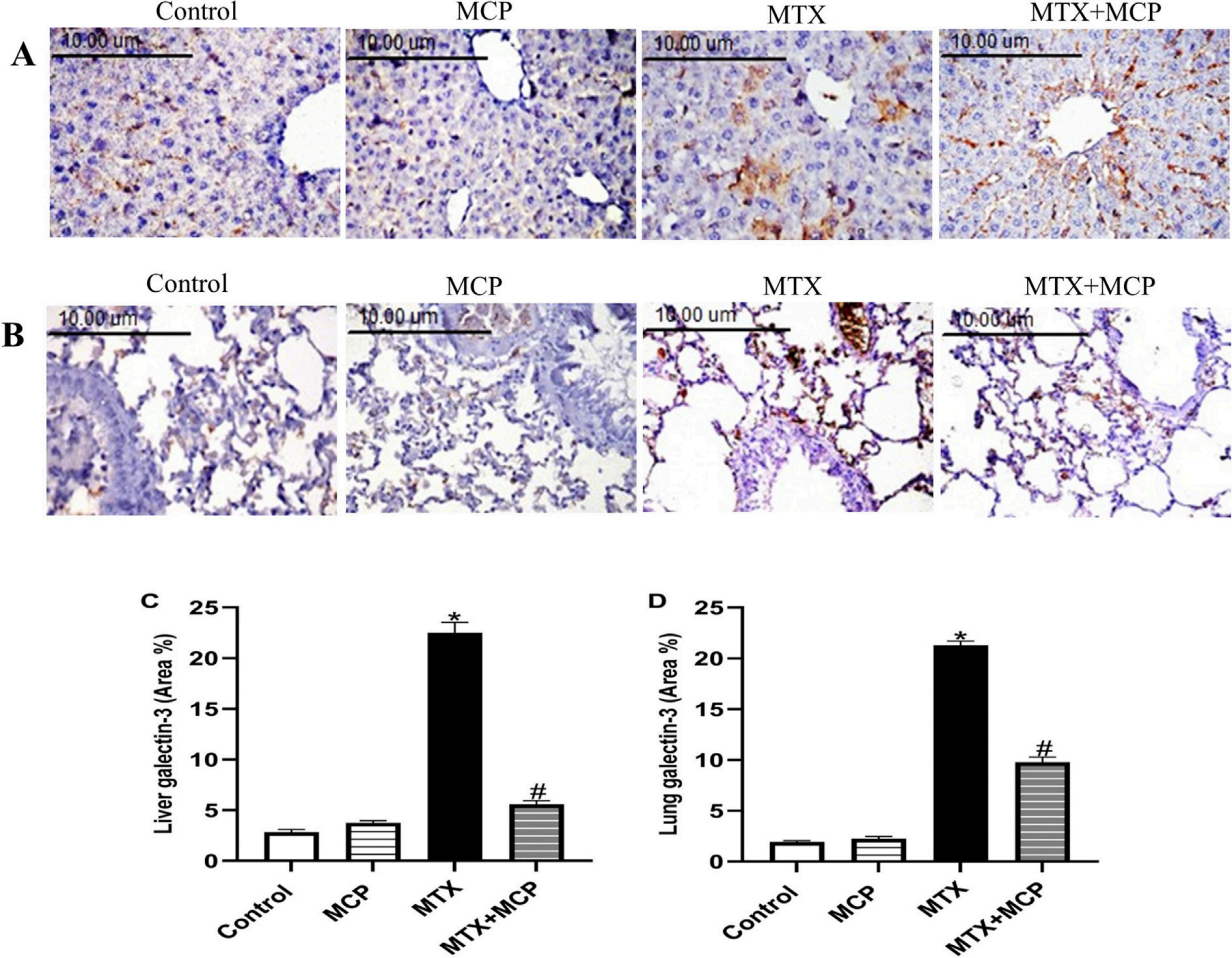
Effect of MTX and MCP on Gal-3 immunoreactivity in liver and lung tissues. **(A)** Photomicrographs representing Gal-3 immunoreactivity in rat liver tissues (X400). **(B)** Photomicrographs representing Gal-3 immunoreactivity in rat lung tissues (X400). **(C)**: The bar chart represents the semi-quantitative analysis of the area percentage of Gal-3 positively stained cells in liver tissues of control, MCP, MTX, and MTX + MCP groups. **(D)** The bar chart represents the semi-quantitative analysis of the area percentage of Gal-3 positively stained cells in lung tissues of control, MCP, MTX, and MTX + MCP groups. Data are expressed as mean ± SEM. Significant differences compared to the control and MTX groups were denoted by * and #, respectively, at *P < 0.05*. MTX: Methotrexate; MCP: Modified citrus pectin; Gal-3: galectin-3.

As illustrated in Figure 5, Gal-3 expression of the control and MCP groups revealed no staining affinity in either liver or lung tissues. On the other hand, MTX showed moderate staining intensity in both investigated tissues. Interestingly, MTX + MCP demonstrated weak staining for Gal-3 expression.

### 3.7 Effect of MTX and MCP on TLR-4 and NF-κB expression in liver and lung tissues

**FIGURE 6:**
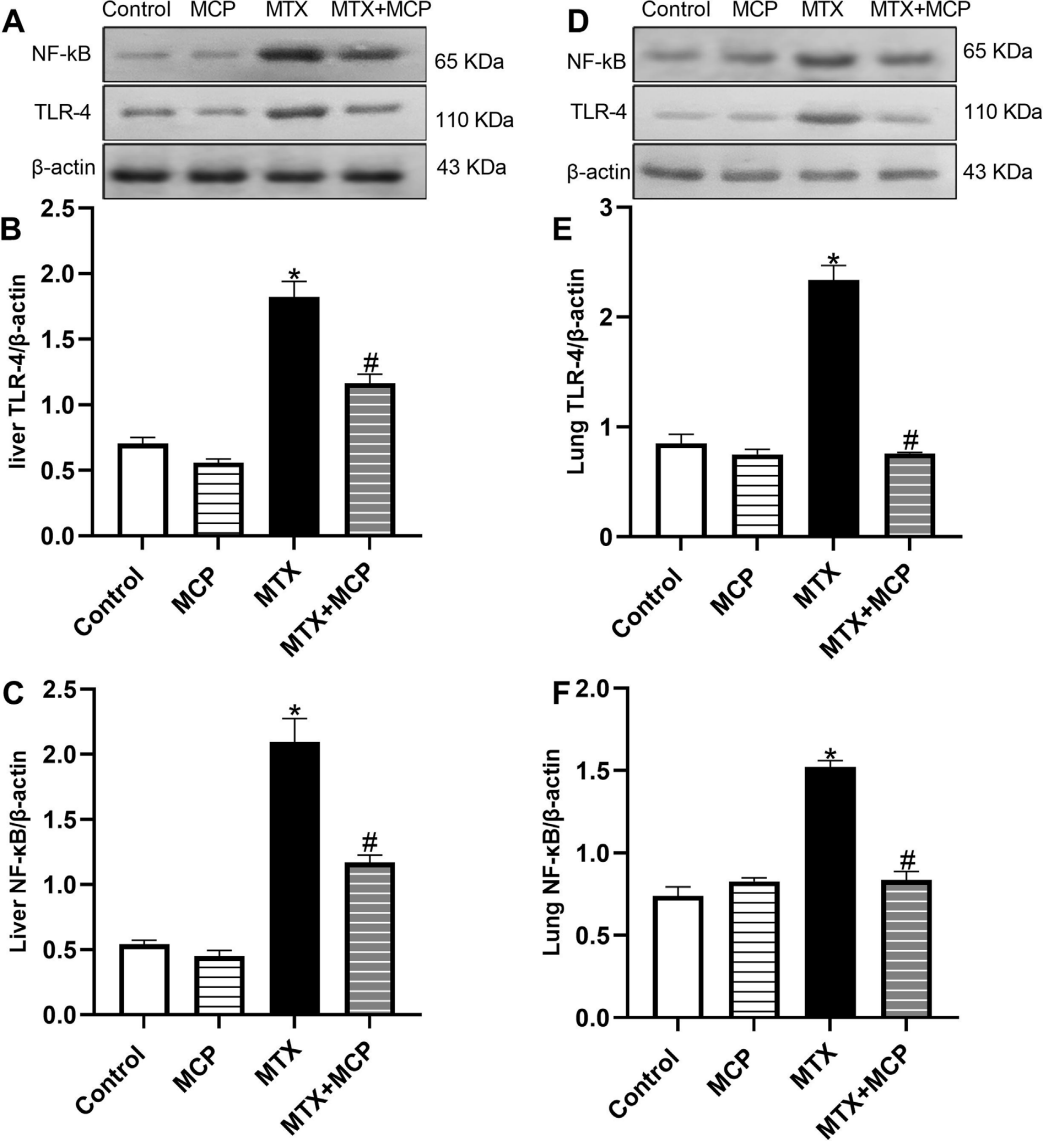
Western blot analysis of the effect of MTX and MCP on TLR-4 and NF-κB expression in liver and lung tissues. **(A, D)** represent TLR-4 and NF-κB bands of control, MCP, MTX, and MTX + MCP groups of liver and lung tissues, respectively. **(B, C)** represent the bar chart of semi-quantitative densitometric analysis of hepatic TLR-4 and NF-κB bands, respectively. **(E, F)** represent the bar chart of semi-quantitative densitometric analysis of pulmonary TLR-4 and NF-κB, respectively. Data are expressed as mean ± SEM. Significant differences compared to the control and MTX groups were denoted by * and #, respectively, at P < 0.05. MTX: Methotrexate; MCP: Modified citrus pectin; NF-κB: Nuclear factor-kappa B; TLR-4: Toll-like receptor-4.

Figure 6 demonstrates how MTX and its combination with MCP affected TLR-4 and NF-κB protein expression, a downstream regulator of TLR-4, in liver and lung tissues. Relative to the control group, MTX resulted in a significant (*P* < 0.05) overexpression of TLR-4 and NF-κB in both tissues. In contrast to the MTX group, cotreatment with MCP significantly (*P*< 0.05) inhibited their increased expression.

### 3.8 Effect of MTX and MCP on TNF-α levels in liver and lung tissues

**FIGURE 7:**
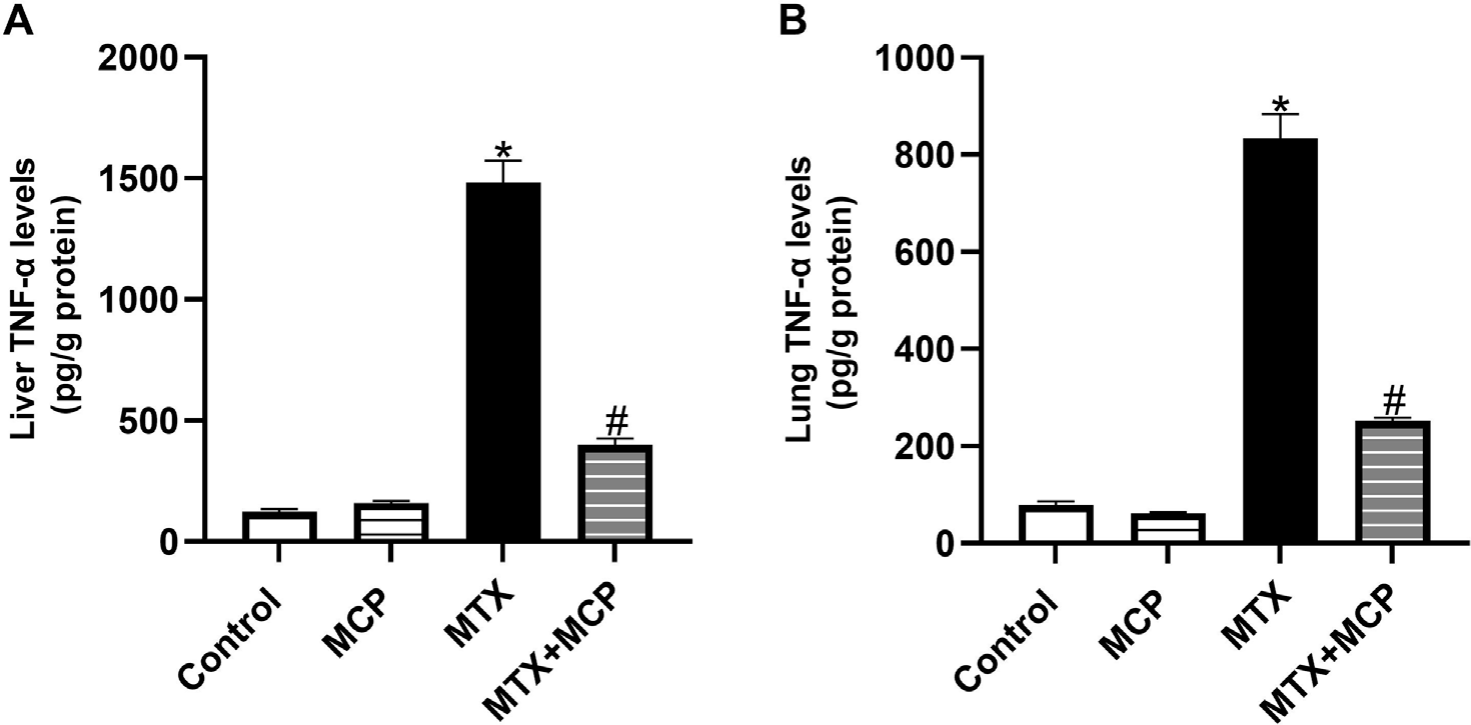
Effect of MTX and MCP on TNF-α levels in liver and lung tissues. **(A)**: Liver TNF-α levels. **(B)**: Lung TNF-α levels. Data are expressed as mean ± SEM. Significant differences compared to the control and MTX groups were denoted by * and #, respectively, at *P < 0.05*. MTX: Methotrexate; MCP: Modified citrus pectin; TNF-α: Tumor necrosis factor-alpha.

As shown in Figures 7A, B, a significant rise of TNF-α levels in both hepatic and lung tissues was observed in the MTX-intoxicated group compared to the control group. Nevertheless, MCP treatment significantly (P < 0.05) halted this increase compared to the MTX group.

### 3.9 Effect of MTX and MCP on TGF-β immunoreactivity in liver and lung tissues

**FIGURE 8:**
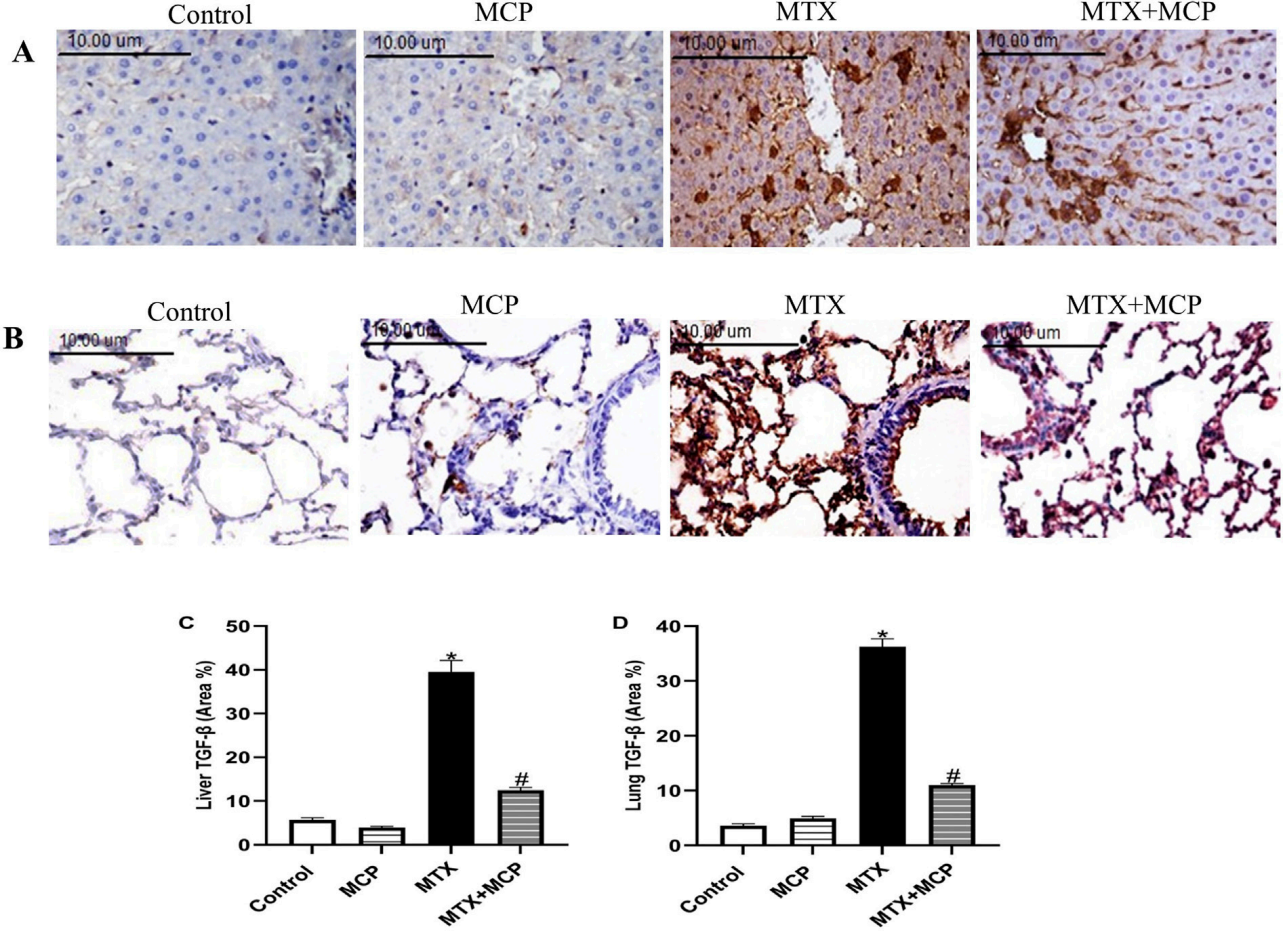
Effect of MTX and MCP on TGF-β immunoreactivity in liver and lung tissues. **(A)** Photomicrographs representing TGF-β immunoreactivity in rat liver tissues (X400). **(B)** Photomicrographs representing TGF-β immunoreactivity in rat lung tissues (X400). **(C)** The bar chart represents the semi-quantitative analysis of area percentage (%) of TGF-β positively stained cells in hepatic tissues of control, MCP, MTX, and MTX + MCP groups. **(D)** The bar chart represents the semi-quantitative analysis of the area percentage (%) of TGF-β positively stained cells in lung tissues of control, MCP, MTX, and MTX + MCP groups. Data are expressed as mean ± SEM. Significant differences compared to the control and MTX groups were denoted by * and #, respectively, at *P < 0.05*. MTX: Methotrexate; MCP: Modified citrus pectin; TGF-β: Transforming growth factor-beta.

No staining affinity to TGF-β was observed in either lung or liver specimens of the control and MCP groups. However, strong staining intensity in these tissues was revealed in the MTX group. It is worth noticing that MCP administration for 14 days significantly attenuated TGF-β expression in both examined tissues relative to the MTX group (Figure 8).

### 3.10 Effect of MTX and MCP on c-caspase-3 expression in liver and lung tissues

**FIGURE 9:**
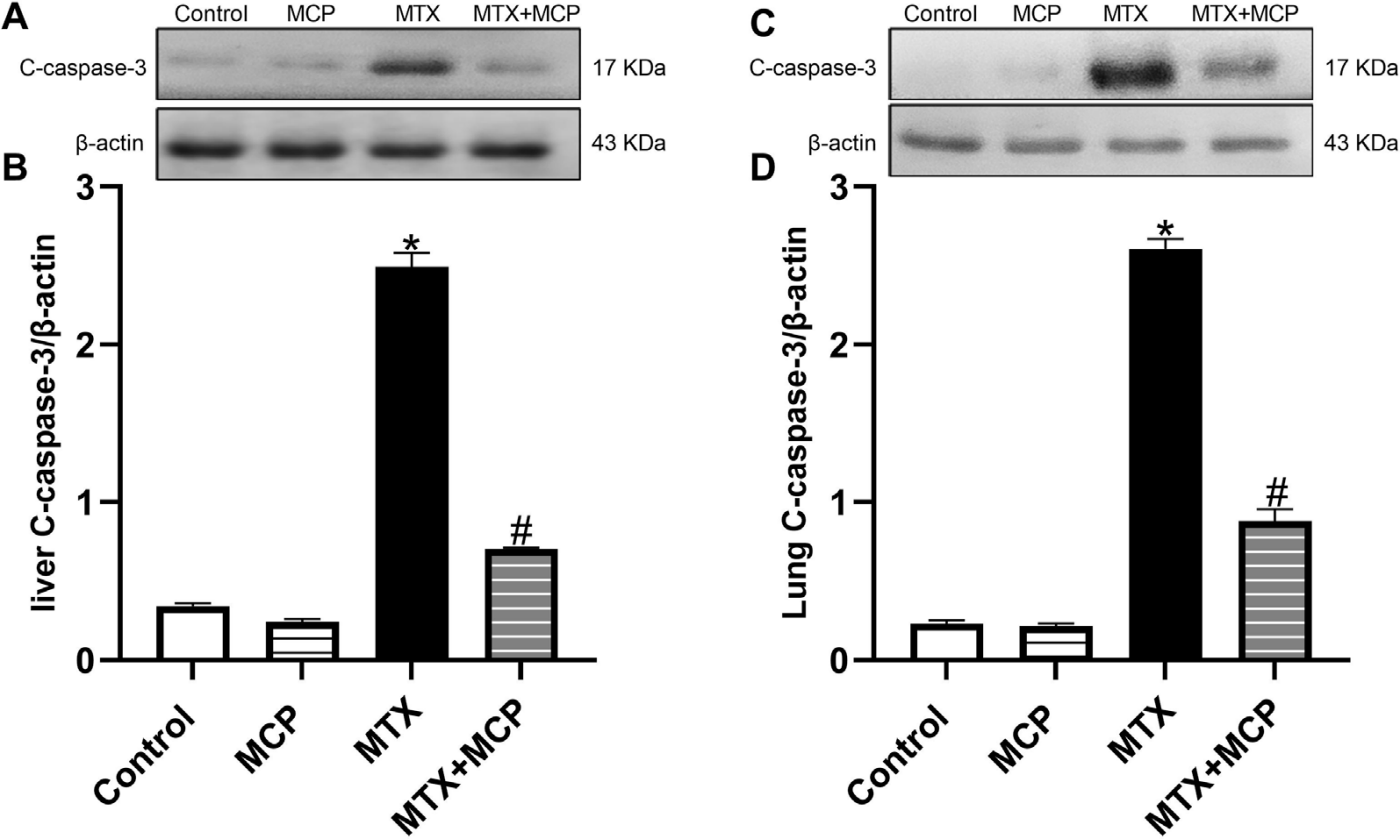
Western blot analysis of the effect of MTX and MCP on c-caspase-3 expression in liver and lung tissues. **(A, C)** represent c-caspase-3 bands of control, MCP, MTX, and MTX + MCP groups of the liver and lung tissues, respectively. **(B, D)** represent the bar chart of semi-quantitative densitometric analysis for both liver and lung tissues, respectively. Data are expressed as mean ± SEM. Significant differences compared to the control and MTX groups were denoted by * and #, respectively, at P < 0.05. MTX: Methotrexate; MCP: Modified citrus pectin; C-caspase-3: Cleaved caspase-3.

As demonstrated in Figure 9, MTX led to a significant (*P* < 0.05) upregulation in the expression of c-caspase-3 in hepatic and lung tissues compared to the control group. However, MCP cotreatment significantly (*P* < 0.05) downregulated MTX-induced overexpression of c-caspase-3 in examined tissues.

### 3.11 Effect of MTX, MCP, and their combination on the viability of MCF7, Nalm6, and JEG3 cells

**FIGURE 10:**
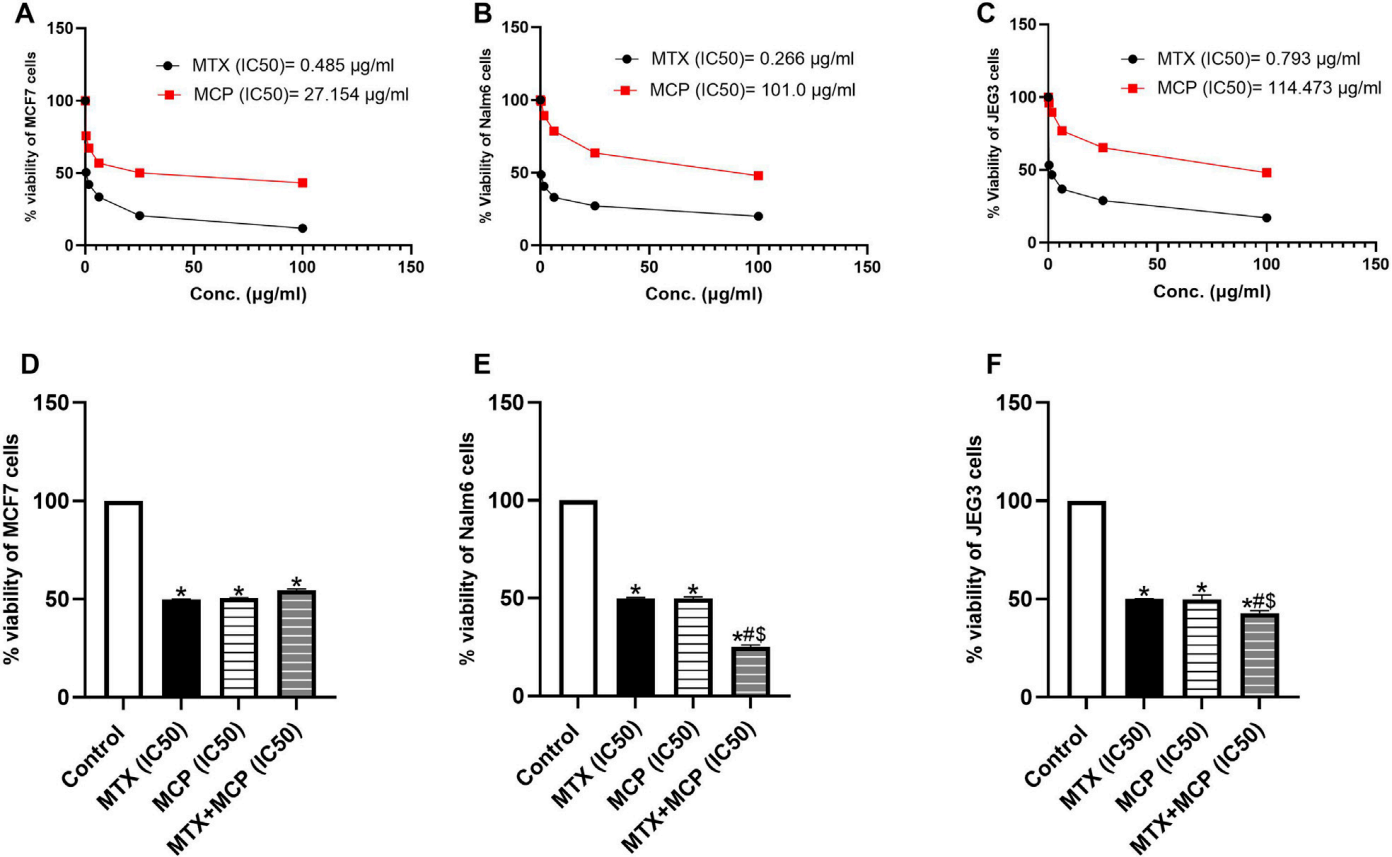
Effect of MTX, MCP, and their combination on the viability of MCF7, Nalm6, and JEG3 at predetermined concentrations. **(A–C)** representing the relation between % cell viability of each cancer cell line and different concentrations of MTX and MCP for determination of IC50 of each drug. **(D–F)** representing the % cell viability of each cell line under the influence of vehicle, IC50 of MTX, IC50 of MCP, and a combination of IC50 of both MTX and MCP. Data are expressed as the mean value of % cell viability± SEM. Significant differences compared to the control, MTX, and MCP groups were denoted by *, #, and $, respectively, at P < 0.05. MTX: Methotrexate; MCP: Modified citrus pectin.

MTX and MCP decreased the cancer cell viability of all cell lines in a concentration-dependent way. Cancer cell viability attenuation was more pronounced in MTX (Figure 10). IC50 values of MTX and MCP were 0.485 and 27.154 μg/mL in MCF7 cells, 0.266 and 101.0 μg/mL in Nalm6 cells, 0.793 and 114.473 μg/mL in JEG3 cells, respectively. The IC50 values of MTX and MCP in each cell line were used to demonstrate whether MCP may affect MTX cytotoxicity. As displayed in Figure 10, the percent cell viability was 50% in all cell lines in the presence of the corresponding IC50 of MTX only. Upon combination with MCP (IC50), the % viability was significantly (*P*< 0.05) reduced in both Nalm6 and JEG3 cells while there was no significant change in MCF7 cells compared to MTX (IC50) alone. Moreover, the % viability of Nalm6 and JEG3 cells was significantly (*P* < 0.05) reduced in the MTX + MCP combination compared to MCP alone. There was no significant change in the % viability of MCF7 cells with the MTX + MCP combination compared to the MCP alone.

## 4 Discussion

Despite being a commonly prescribed chemotherapeutic and immunosuppressant agent (Pivovarov and Zipursky, 2019; Koźmiński et al., 2020), MTX use is limited due to several adverse effects such as liver and lung toxicity which represent a major clinical challenge (Kim et al., 2009; Ezhilarasan, 2021). For the first time, we reported the potential protective effects of MCP against MTX-elicited liver and lung toxicity in rats as evidenced by improving markers of liver and lung functions and restoring normal liver and lung structure. Mitigating oxidative stress, inflammation, fibrosis, and apoptosis might also contribute to MCP’s therapeutic impact.

Consistent with previous experimental and clinical studies (Ali et al., 2014; Mori et al., 2018; Cao et al., 2019; Karlsson Sundbaum et al., 2019; Roghani et al., 2020), MTX-induced hepatotoxicity was presented through a pronounced elevation in serum ALT and AST levels. Besides the damaged liver histological structure; disorganized hepatic cords, hepatocyte ballooning degeneration with nuclear pyknosis, and hepatic apoptosis (Mahmoud et al., 2017a; Al Kury et al., 2020). The elevated serum liver enzymes may be ascribed to hepatocellular degeneration, loss of hepatocytes structural integrity, and leak of their contents into the blood (McGill, 2016; Rizk et al., 2018).

The MCP hepatoprotective effect against MTX was verified here by the decline in the elevated serum ALT and AST levels alongside the improved liver architecture. As reported before (Kelleni et al., 2016; Kalantari et al., 2019), MTX led to hepatomegaly in rats which was attenuated, here, by MCP. MCP hepatoprotective influence was reported in CCl4-induced liver fibrosis in rats (Abu-Elsaad and Elkashef, 2016).

Regarding MTX-caused pulmonary toxicity, the relative lung weight and lung W/D weight ratio in addition to total protein content and leukocyte count in BALF were notably elevated in the MTX group which are features of acute lung injury (Abraham, 2003; Poitout-Belissent et al., 2021) consistent with previous studies (Rajizadeh et al., 2023; Rajizadeh et al., 2024). The BALF analysis of patients with MTX-induced pneumonitis revealed lymphocytosis and elevated neutrophil counts (D'Elia, 2014). Lymphocyte proliferation and hypersensitivity pneumonitis triggered by alveolitis are linked to cellular immune response and cytokine release (Kim et al., 2009). The histopathological findings showed lymphocytes and macrophage infiltration, disrupted lung architecture, thickened inter-alveolar septa, and capillary congestion as reported before (Arpag et al., 2018; Ozmen et al., 2024).

Here, MCP ameliorated lung edema indices, BALF total protein content, and leukocyte count, and improved the histological architecture, suggesting its protective effects against MTX-induced lung toxicity.

Despite no obvious mechanism illustrating MTX-induced organ dysfunction, accumulation of MTX polyglutamate inside hepatocytes, the metabolized form of MTX, has been reported as the key factor of MTX-induced hepatotoxicity. It decreases the folic acid reservoir which consequently induces several pathological events associated with oxidative stress, inflammation, apoptosis, and fibrosis (Hawwa et al., 2015; Yamamoto et al., 2016).

The impairment of the tissue oxidant/antioxidant balance remains the hallmark cause of MTX-induced organ toxicity resulting in oxidative damage (Gao and Horie, 2002; Huang et al., 2005; Chang et al., 2013). This oxidative damage of protein and DNA in addition to lipid peroxidation causes disarrangement of the lipid bilayer membrane, deactivation of membrane-bound receptors and enzymes, and, in turn, enhances tissue permeability (Halliwell and Gutteridge, 2015; Malayeri et al., 2022) which can explain the elevated liver enzymes with MTX (Dalaklioglu et al., 2013).

The pathogenesis of MTX-induced hepatotoxicity (Cetin et al., 2008; Çakır et al., 2011; Pınar et al., 2018; Kalantar et al., 2019) and lung toxicity (Kurt et al., 2015; Saygin et al., 2016; Arpag et al., 2018) is known to involve oxidative stress. An elevation in MDA levels, a marker of lipid peroxidation, and a decrease in GSH levels as well as inhibition of SOD antioxidant activity, in liver and lung tissues were demonstrated in the MTX group consistent with earlier studies (Demiryilmaz et al., 2012; Mohamed et al., 2019; Hussein et al., 2020; Goudarzi et al., 2021; Zaki et al., 2021; Parthasarathy and Prince, 2023; Abdalhameid et al., 2024). GSH reduction was explained previously by MTX inhibitory effect on cytosolic reduced nicotinamide adenine dinucleotide phosphate (NADPH) (Vardi et al., 2010) which is required for GSH maintenance by glutathione reductase (Ali et al., 2017).

Conversely, MCP significantly rebalanced the oxidative status in both tissues, indicating its antioxidant properties. The antioxidant activity of MCP was revealed in various animal studies including diabetes-associated cognitive impairment (Yin et al., 2020), CCl4-induced liver fibrosis (Abu-Elsaad and Elkashef, 2016), doxorubicin-induced cardiotoxicity (Tian et al., 2020), diabetes-induced nephropathy (Mahmoud et al., 2024), diet-induced obesity (Marín-Royo et al., 2018), and *in vitro* model of mouse monocytes (Ramachandran et al., 2017).

The master and emerging regulator of cellular antioxidant defense, Nrf2, induces the transcription of antioxidant enzymes and enzymes involved in GSH and NADPH regeneration (Ma, 2013). Although Nrf2 is activated during oxidative stress, the generation of huge amounts of reactive oxygen species (ROS) suppresses its expression (Mahmoud et al., 2017a; Mahmoud et al., 2017b). In agreement with other studies (Mukherjee et al., 2013; Bu et al., 2018; Fayez et al., 2018; Kawami et al., 2022), MTX greatly downregulated Nrf2 expression in hepatic and pulmonary tissues parallel to the findings of MDA, SOD, and GSH. Conversely, MCP prevented Nrf2 downregulation which, subsequently, reduced oxidative stress and improved antioxidant defense. These antioxidant effects of MCP can play a significant role in its protective effects against MTX-induced hepatic and pulmonary toxicity.

Galectin-3 is pivotal in fibrosis and inflammation (Mackinnon et al., 2012; An et al., 2021; Slack et al., 2021; Boutin et al., 2022; Lima et al., 2023). As a proinflammatory protein, Gal-3 initiates and amplifies acute inflammatory response through the recruitment of macrophages to the injury site and perpetuating a chronic inflammatory state through the induction of proinflammatory pathways (Bouffette et al., 2023). The embroilment of inflammation was documented in the pathogenesis of MTX-induced liver (Ali et al., 2017; Mahmoud et al., 2017a; Al Kury et al., 2020) and lung (Mammadov et al., 2019; Zaki et al., 2021; Ozmen et al., 2024) toxicity. This study aimed to demonstrate the significance of Gal-3, as a therapeutic target, in MTX-induced liver and lung toxicity, which has yet to be explored, using MCP as a natural Gal-3 inhibitor.

Galectin-3 was reported as an endogenous paracrine ligand and activator of TLR-4 inducing an inflammatory response (Burguillos et al., 2015). TLR-4, a member of the pattern recognition receptors, is an important sensor of the innate immune response that can interact with exogenous molecules such as lipopolysaccharide (LPS) of Gram-negative bacteria which are recognized as pathogen-associated molecular patterns. Additionally, it can be triggered by endogenous molecules of damaged or necrotic cells such as heat shock protein after oxidative stress which are recognized as damage-associated molecular patterns (Lu et al., 2008; Gill et al., 2010). This interaction eventually leads to an inflammatory cascade through the activation of NF-κB and elevated transcription of proinflammatory cytokines such as TNF-α (Miller et al., 2005).

Recently, the contribution of the TLR-4/NF-κB signaling pathway in MTX-induced liver toxicity was documented (Matouk et al., 2022; Manna et al., 2023). To the best of our knowledge, its importance in MTX-induced lung toxicity has yet to be established. Here, high expression of Gal-3 along with TLR-4/NF-κB/TNF-α signaling pathway upregulation was observed in the hepatic and lung tissues of the MTX group. The activation of TLR-4/NF-κB signaling in LPS-caused injury in chondrocytes mediated the inflammatory and proapoptotic actions of Gal-3 while Gal-3 silence resulted in apoptosis inhibition through inhibition of the inflammatory response (Wang et al., 2019).

Crosstalk between TLR-4 and oxidative stress has also been reported. ROS may lead to TLR-4 activation which, in turn, may increase ROS production through direct interaction between NADPH oxidase and TLR-4 (Gill et al., 2010). Reports discussing relation between NF-κB and Nrf2 revealed that Nrf2 is a negative regulator of NF-κB signaling dampening NF-κB activation as well as limiting the transcription and overproduction of proinflammatory cytokines (Ahmed et al., 2017). Moreover, Nrf2 activation may mitigate TLR-4-induced inflammation under pathological conditions (Huang et al., 2014; Marinovic et al., 2015). This crosstalk may also be a result of the Nrf2/NF-κB relationship (Wardyn et al., 2015; Mohan and Gupta, 2018). In a model of ischemic-reperfusion liver injury, it was stated that induction of Nrf2 attenuated TLR-4-induced liver inflammation and ameliorated oxidative stress (Huang et al., 2014).

We found that the MCP-induced blockade of Gal-3 protected against MTX-induced liver and lung toxicity by suppressing TLR-4 and its downstream regulator, NF- κB. Hence, one of the critical mediators of inflammation and apoptosis was decreased, TNF-α. Previous studies indicated that the MCP-induced Gal-3 inhibition exhibited anti-inflammatory effects in animal models analyzing erectile dysfunction (Wang et al., 2024), cerebral-ischemia reperfusion injury (Cui et al., 2022), and myocardial fibrosis (Xu et al., 2020) via the downregulation TLR-4/NF-κB signaling pathway. Collectively, the inhibition of the TLR-4/NF-κB/TNF-α signaling by MCP could be explained by both inhibition of Gal-3 and activation of Nrf2. The anti-inflammatory effects of MCP can explain, to a certain extent, its protective effects against MTX toxicity.

Unresolved inflammation and abnormal tissue repair can result in tissue fibrosis (Bouffette et al., 2023). As a profibrotic protein, Gal-3 is identified as a biomarker for the progression of fibrosis (Li et al., 2014) and its expression was increased in the bleomycin-caused pulmonary fibrosis mouse model and patients with idiopathic pulmonary fibrosis (Nishi et al., 2007; Mackinnon et al., 2012) or liver fibrosis (Mackinnon et al., 2023). MTX-elicited fibrosis in the liver (Taskin et al., 2017; Cao et al., 2019; Ahmad et al., 2021) and lung (Saygin et al., 2016; Abdalhameid et al., 2024; Manie et al., 2024) tissues were identified. In this work, MTX led to elevated expression of TGF-β, a profibrotic cytokine. TGF-β stimulation eventually activates tissue fibroblasts into active myofibroblasts leading to extracellular matrix synthesis (Biernacka et al., 2011; Meng et al., 2016). TGF-β is significant in MTX-induced pulmonary fibrosis which is at least partially mediated by epithelial-mesenchymal transition (EMT) at which myofibroblasts originate from the injured epithelial cells (Ohbayashi et al., 2014). Moreover, MTX-induced downregulation and reduced activity of Nrf-2 are also involved in MTX-caused EMT in alveolar epithelial cell lines (Kawami et al., 2022). Gal-3 has an important role in the regulation of EMT induction (Mackinnon et al., 2012).

Gal-3 is mandatory for TGF-β-induced myofibroblast activation and extracellular matrix production (Henderson et al., 2006). Gal-3 has recently been shown to activate TGF-β in human pulmonary fibroblasts and its inhibition can prevent TGF-β activation (Calver et al., 2024). Moreover, it was identified that the pharmacological inhibition of Gal-3 in the NAFLD mice model downregulated TGF-β (Lee et al., 2022). MCP counteracted the profibrotic effects of Gal-3 in different disease models (Kolatsi-Joannou et al., 2011; Calvier et al., 2013; Martínez-Martínez et al., 2015; Vergaro et al., 2016; Li et al., 2018; Ibarrola et al., 2019; Yin et al., 2020). Consistent with previously mentioned findings, MCP-induced Gal-3 inhibition downregulated hepatic and pulmonary TGF-β in MTX-treated rats. So, the inhibition of the fibrotic Gal-3/TGF-β pathway by MCP can protect against MTX-induced liver and lung fibrosis.

Hepatic and pulmonary fibrosis induced by MTX were additionally confirmed by Masson’s trichrome staining which showed the proliferation of fibrous tissue, as previously reported (Tag, 2015; Mohamed et al., 2019; Abdalhameid et al., 2024). However, MCP treatment decreased collagen deposition in both liver and lung tissues in line with the results of Gal-3 and TGF-β. Similarly, MCP decreased collagen content demonstrated by Masson’s trichrome staining in the cisplatin-induced nephrotoxicity model (Li et al., 2018).

Several studies reported that the induction of apoptosis may mediate MTX-induced liver (Mahmoud et al., 2017a; Khafaga and El-Sayed, 2018; Türk et al., 2022) and lung (Kurt et al., 2015; Abosrea et al., 2023; Ozmen et al., 2024) damage. Consistent with previous reports (Ali et al., 2014; Rajizadeh et al., 2024), MTX caused c-caspase-3 overexpression in both examined tissues which can be related to the elevated levels of ROS and proinflammatory cytokines (Simon et al., 2000; Jaeschke, 2011). It is worth mentioning that Gal-3 may have a function in regulating apoptosis. It may act as an antiapoptotic factor due to its sequence homology to B-cell lymphoma 2 (Bcl-2), an apoptosis suppressor (Yang et al., 1996). However, a previous study showed that extracellular Gal-3 acts as a proapoptotic factor triggering apoptosis in activated T-cells leading to mitochondrial apoptosis involving the release of cytochrome c and activation of caspase-3 (Fukumori et al., 2003). In the present, MCP showed antiapoptotic effects through downregulating c-caspase-3 which may result from the Gal-3 inhibition, anti-inflammatory and antioxidant influences of MCP parallel to earlier studies reporting the antiapoptotic properties of MCP (Abu-Elsaad and Elkashef, 2016; Li et al., 2018; Tian et al., 2020; Mahmoud et al., 2024).

Galectin-3 has a tumor-promoting effect in different tumors (Eliaz and Raz, 2019). It promotes cancer cell resistance to chemotherapeutic agents acting as a potent inhibitor of the intrinsic apoptosis pathway (Nakahara et al., 2005; Fukumori et al., 2007; Navarro et al., 2020). Several previous reports have shown that MCP, through Gal-3 inhibition, modulates multiple rate-limiting steps of cancer metastasis (Glinsky and Raz, 2009; Eliaz and Raz, 2019). The anticancer activity of MCP was demonstrated before such as in the colon (Liu et al., 2008; Wu et al., 2018), prostate (Yan and Katz, 2010), ovarian (Hossein et al., 2013), and breast (Garrido et al., 2024) cancers. According to these findings, MCP may be used as a natural chemosensitizer with chemotherapeutic agents (Chauhan et al., 2005; Johnson et al., 2007). Accordingly, our findings of the MTT cell viability assay showed that both MTX and MCP significantly reduced, in a concentration-dependent way, the cell viability of MCF7, Nalm6, and JEG3 at which the expression of Gal-3 in these cell lines was previously documented (Simone et al., 2014; Jovanović et al., 2024; Li et al., 2024). Moreover, combining MCP (IC50) with MTX (IC50) enhanced MTX cytotoxicity efficacy by decreasing cell viability by less than 50% compared to MTX alone in Nalm6 and JEG3 cells. The MCP-induced inhibition of Gal-3 could demonstrate this.

In conclusion, this study demonstrates that MCP protects against MTX-caused hepatic and pulmonary toxicity through anti-inflammatory, antiapoptotic, antifibrotic, and antioxidant properties. The antioxidant effects are evidenced by the upregulation of Nrf2 expression, a decrease in MDA levels, and an increase in SOD activity and GSH levels. Moreover, MCP downregulated the inflammatory signaling pathway Gal-3/TLR-4/NF-κB pathway. Additionally, MCP decreased c-caspase-3, TGF-β, and collagen levels in liver and lung tissues. These effects were reflected in the improved liver and lung functional markers and histological structure. Moreover, the cytotoxicity of MTX was enhanced by MCP in different human cell lines. A limitation of the study is the necessity for *in vitro* and further *in vivo* studies to investigate additional protective mechanistic pathways of MCP against MTX-induced hepatic and pulmonary toxicity.

The study outcomes are significant clinically where MTX is commonly prescribed. As a natural product, MCP can be combined with MTX treatment protocols to decrease the incidence and severity of its associated adverse effects thus enhancing the patient’s outcome. Clinical trials are required before practical application to assess efficacy, safety, appropriate dosage, and time required for pretreatment to offer its protective effects.

## Data Availability

The datasets presented in this study can be found in online repositories. The names of the repository/repositories and accession number(s) can be found in the article/supplementary material.
